# Evaluating the impact of modeling the family effect for clonal selection in potato-breeding programs

**DOI:** 10.3389/fpls.2023.1253706

**Published:** 2023-10-30

**Authors:** Vinicius Samuel Martins, Mario Henrique Murad Leite Andrade, Leticia Novais Padua, Luciana Aparecida Miguel, Claudio Carlos Fernandes Filho, Marcio Lisboa Guedes, Jose Airton Rodrigues Nunes, Leo Jr Hoffmann, Lincoln Zotarelli, Márcio Fernando Ribeiro de Jr Resende, Pedro Crescêncio Souza Carneiro, Tiago de Souza Marçal

**Affiliations:** ^1^ Departamento de Biologia, Universidade Federal de Lavras, Lavras, Brazil; ^2^ School of Food and Agriculture, University of Maine, Orono, ME, United States; ^3^ Centro de Tecnologia Canavieira (CTC), Piracicaba, Brazil; ^4^ Rede Interuniversitária para o Desenvolvimento do Setor Sucroenergético (RIDESA), Universidade Federal de Goiás, Goiânia, Brazil; ^5^ Department of Horticultural Sciences, Institute of Food and Agricultural Sciences, University of Florida, Gainesville, FL, United States; ^6^ Departamento de Biologia, Universidade Federal de Viçosa, Viçosa, Brazil

**Keywords:** *Solanum tuberosum* L., nested structure, accuracy, G×E interactions, autotetraploid genetics, tuber yield

## Abstract

Because of its wide distribution, high yield potential, and short cycle, the potato has become essential for global food security. However, the complexity of tetrasomic inheritance, the high level of heterozygosity of the parents, the low multiplication rate of tubers, and the genotype-by-environment interactions impose severe challenges on tetraploid potato–breeding programs. The initial stages of selection take place in experiments with low selection accuracy for many of the quantitative traits of interest, for example, tuber yield. The goal of this study was to investigate the contribution of incorporating a family effect in the estimation of the total genotypic effect and selection of clones in the initial stage of a potato-breeding program. The evaluation included single trials (STs) and multi-environment trials (METs). A total of 1,280 clones from 67 full-sib families from the potato-breeding program at Universidade Federal de Lavras were evaluated for the traits total tuber yield and specific gravity. These clones were distributed in six evaluated trials that varied according to the heat stress level: without heat stress, moderate heat stress, and high heat stress. To verify the importance of the family effect, models with and without the family effect were compared for the analysis of ST and MET data for both traits. The models that included the family effect were better adjusted in the ST and MET data analyses for both traits, except when the family effect was not significant. Furthermore, the inclusion of the family effect increased the selective efficiency of clones in both ST and MET analyses via an increase in the accuracy of the total genotypic value. These same models also allowed the prediction of clone effects more realistically, as the variance components associated with family and clone effects within a family were not confounded. Thus, clonal selection based on the total genotypic value, combining the effects of family and clones within a family, proved to be a good alternative for potato-breeding programs that can accommodate the logistic and data tracking required in the breeding program.

## 1 Introduction

Potato is the third most important crop for human consumption worldwide, playing a central role in global food security. Potato has a wide adaptation and higher yield compared with cereal crops (FAO, 2020). It will certainly continue to have an essential role in food security in the coming years, particularly regarding population growth (Devaux et al., 2014; FAO, 2020; Devaux et al., 2021). The world’s average potato production has grown at a rate of 2% per year for the past 20 years, with an average yield of 21.0 Mg ha^−1^, which represents only 13% of the potential yield (Kunkel and Campbell, 1987; FAO, 2021). The gap in production between average and potential yield presents the potential for increasing global potato production. This potential can be exploited through technological innovations in the potato production system, using new and improved cultivars, and optimizing agricultural practices. Within the framework of genetic improvement, tuber yield can be increased through an accurate selection of clones more tolerant to biotic and abiotic stresses, and more efficient in the use of resources, such as water and nitrogen, meeting the demand for an increasingly sustainable global production system (Foley et al., 2011; Birch et al., 2012; Obidiegwu et al., 2015; Dahal et al., 2019; Devaux et al., 2021).

Tetraploid potato–breeding programs generate thousands of seedlings annually (Stich and Van Inghelandt, 2018). Larger populations are required to increase the probability of selecting superior clones because potato breeders must deal with the complexity of tetrassomic segregation, heterosis, and high level of heterozygosity of parents (Meyer et al., 1998; Gopal, 2015). A small number of seed potatoes are available in the early stages of a potato-breeding program (Haynes et al., 2012; Stich and Van Inghelandt, 2018), which restricts the use of repetitions and the number of plants per plot (Haynes et al., 2012; Paget et al., 2017). In this context, the use of unreplicated designs, such as the augmented block design (ABD) (Federer, 1956), has been frequent (Andrade et al., 2020; Fernandes Filho et al., 2021). Partially replicated design (P-REP) (Cullis et al., 2006) is an efficient alternative in the initial stages of potato breeding (Paget et al., 2017). Furthermore, P-REP can be increased (Williams et al., 2011; Williams et al., 2014), i.e., a proportion of candidates can be replicated in each location, which allows the study of genotype-by-environment interaction (G×E) even with limited seed.

The G×E heavily influences quantitative traits of economic importance in potatoes. Currently, up to 40 traits can be selected in potato-breeding programs (Bradshaw, 2017), where the low correlation of the main traits between the environments results in a considerable loss of genetic gain (Andrade et al., 2021). This effect is significant for potato-breeding programs in tropical and subtropical regions as the crop is grown in different seasons throughout the year (winter, fall, and summer). Therefore, one goal of this program is the development of heat-tolerant clones by assessing promisor clones in contrasting seasons to determine their ability to withstand heat stress (Fernandes Filho et al., 2021; Patiño-Torres et al., 2021). Mixed model methodologies have an important role in connecting these different experiments and estimating parameters that are useful for the selection process (Henderson et al., 1959; Smith et al., 2001; Smith et al., 2015).

The limited number of repetitions, presence of G×E, and low heritability result in low selection accuracy in the early stages of a potato-breeding program. This implies low genetic progress over the selection cycles once accuracy is directly proportional to expected gains with selection (Cobb et al., 2019). Using a genetic relationship matrix has been demonstrated to increase the accuracy of estimated breeding values for traits with low heritability. This approach leverage information from all relatives (half and full-sibs, parents, etc.) to accurately estimate the breeding values of candidates (Slater et al., 2014a). However, in estimating non-additive effects, such as dominance, you need a balanced mating design to capture general and specific combining ability (Amadeu et al., 2020; Voss-Fels et al., 2021; Yadav et al., 2021).

The prediction of the total genotypic value (additive + non-additive effects) for complex quantitative traits, such as tuber yield, in the initial stage of the potato-breeding programs, relies on the use of genomic resources (Stich and Van Inghelandt, 2018; Amadeu et al., 2020; Voss-Fels et al., 2021; Wilson et al., 2021; Yadav et al., 2021). Nevertheless, early-stage genotyping is more expensive than phenotyping, making unfeasible use of genomic selection in some research, especially in stages where many candidates were evaluated (Stich and Van Inghelandt, 2018; Wilson et al., 2021; Bradshaw, 2022). Alternatively, Piepho et al. (2008) argued that the use of models with nested structure (Family/Clone = Family + Family  +  Clone) could be advantageous. These models account implicitly for the kinship relationship. Furthermore, using this structure allows us to predict the total genotypic value more easily, without the need for a kinship matrix, which can be valuable in cases where the mating design does not allow an accurate estimation of the specific combining ability.

The dominance effect can be estimated with a kinship matrix using complete mating design or using genomics. A third alternative is the modeling of the family effect. Although the nested structure appears naturally in the initial stage of potato-breeding programs because seedlings are derived from different crosses (usually bi-parental), the family effect has been neglected (Fernandes Filho et al., 2021), mainly due to the easiness of mass selection in the early stages of selection. Therefore, it is hypothesized that using nested structure models can increase the selective efficiency of clones, both in single-trial (ST) and multi-environment–trial (MET) selection schemes.

Thus, this work aims to investigate the impact of the family effect and the selection accuracy of clones in the initial stage of a tetraploid potato–breeding program in ST and MET clonal selection schemes.

## 2 Materials and methods

### 2.1 Field trials

#### 2.1.1 Experimental designs and crop management

A total of six trials from the potato-breeding program at the Universidade Federal de Lavras (PROBATATA-UFLA) were installed at the Center for Scientific and Technological Development, City Lavras, Minas Gerais State, Brazil (21°12′19.8″S, 44°58′48.8″W), located at 919 m American Sign Language (ASL), and soil was classified as red-yellow latosol.

The details of each trial are shown in **Table 1**. Four trials were designed in an ABD (Federer, 1956), and two trials a P-REP with *p_N_* around 20% were employed (Cullis et al., 2006).

**Table 1:** Trials characterization, size, and number of blocks, family, clone, check, and percentage of plots replicate.

Trial^†^	Year	Design^‡^	Number of levels					*p_N_* ^§^
			Block	Family	Clone	Check	Plot	
POP1(WHS)	2013	ABD	48	24	477	3	621	22.71
POP2(WHS)	2017	ABD	20	31	491	2	531	7.16
POP2(MHS)	2017	ABD	20	31	491	2	531	7.16
POP2(HHS)	2018	ABD	20	31	491	2	531	7.16
POP3(WHS)	2021	P-REP	20	12	304	4	400	23.00
POP3(HHS)	2021	P-REP	20	12	312	3	400	21.25

**^†^** The trial identification: POP1(WHS), POP2(WHS), POP2(MHS), POP2(HHS), POP3(WHS), and POP3(HHS), where the codes POP1, POP2, and POP3 identify different clonal populations and codes WHS, MHS, and HHS identify three different seasons, varying in the function of stress level: without heat stress (WHS) moderate heat stress (MHS), and high heat stress (HHS).

**^‡^** Experimental designs: augmented block design (ABD) and partially replicated design (P-REP).

^§^p_N_: percentage of plots experimental units occupied by replicated clones, given by the expression. p_N_ = (N − N_treat_)/N, where N is the number of plots and N_treat_ is the number of treatments.

Each plot consisted of five plants spaced 0.30 m between plants and 0.80 m between rows. Crop management practices for all the trials were done according to the recommendations for the state of Minas Gerais, in which 1.5 Mg ha^−1^ of 08-28-16 fertilizer blend (N–P_2_O_5_–K_2_O) was applied during the planting. Side dress fertilizer application was performed with 0.30 Mg ha^−1^ 20-00-20 (N–P_2_O_5_–K_2_O). All the trials were irrigated using a sprinkler irrigation system, according to the need of the crop and the incidence of rainfall through the seasons.

#### 2.1.2 Levels of heat stress

**Figure 1:**
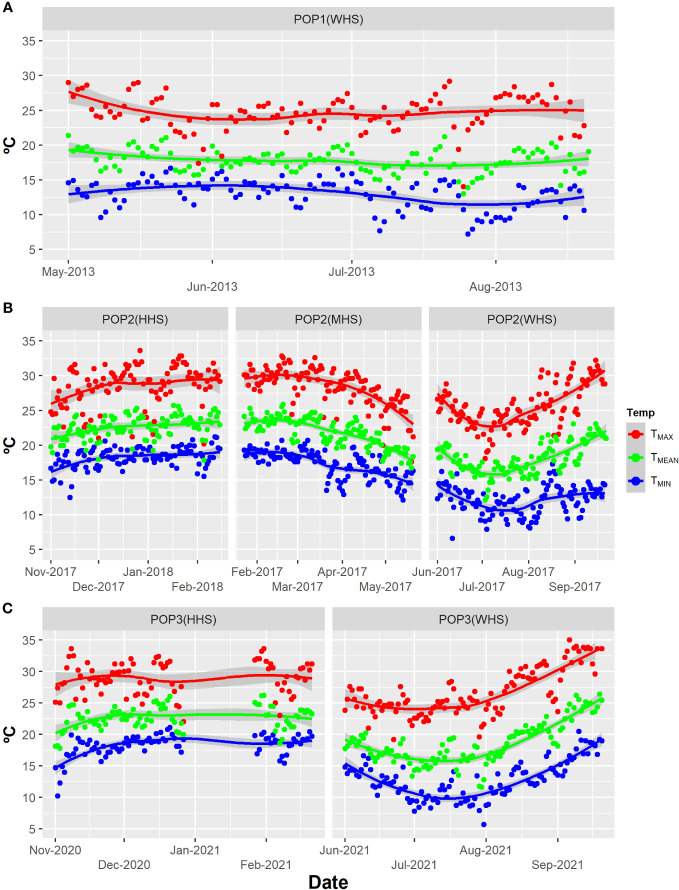
Temp: Maximum temperature (T_MAX_), minimum temperature (T_MIN_), and compensated mean temperature (T_MEAN_) in degrees Celsius for all trials: **(A)** POP1(WHS), ranging from May to August of 2013; **(B)** POP2(WHS), ranging from June to September 2017; POP2(MHS), ranging from February to May 2017; POP2(HHS), ranging from November 2017 to February 2018; POP3(WHS), ranging from June to September 2021; and **(C)** POP3(HHS), ranging from November 2020 to February 2021 for Lavras, Minas Gerais State, Brazil (INMET, 2022). Seasons varying in terms of stress level: without heat stress (WHS) moderate heat stress (MHS), and high heat stress (HHS) from 2013 to 2021.

The trials were evaluated in three seasons with different levels of heat stress: without heat stress (WHS), moderate heat stress (MHS), and high heat stress (HHS) (**Figure 1**). The trials WHS (season from May to September), MHS (February to May), and HHS (November to February) were carried out during winter, fall, and summer seasons, respectively (**Figure 1**; **Table 1**).

The compensated mean temperatures (TMEAN, °C) were obtained using the expression TMEAN = (T9am + 2T9pm + TMAX + TMIN)/5, where T9am, T9pm, TMAX, and TMIN are the air temperature at 9 a.m., 9 p.m., maximum, and minimum, respectively (INMET, 2022).

#### 2.1.3 Phenotyping

Two traits were evaluated in each trial: total tuber yield (TTY − Mg ha^−1^) and specific gravity (SG). Total tuber yield was estimated by the weight of all tubers harvested in 1.2 m^2^ for each plot. The SG was estimated by the expression SG = tuber mass in air/(tuber mass in the air − tuber mass in water), where tuber mass in air and tuber mass in water were measured from fresh samples of tubers, ranging from 2.0 kg and 2.5 kg, using a hydrostatic scale (Schippers, 1976).

### 2.2 Statistical analysis

A nested genetic treatment structure was evaluated. The clones were obtained from different clonal families. Thus, it is possible to access family and clone within-family effects from the data.

For the analysis, in which *c* clones were sampled from *s* clonal families and evaluated together with *p* checks, the general form of the linear mixed model is presented in Equation (1). This model is suitable for both ST or MET data. For MET data analysis, appropriate (co)variance structures should be used to model the vectors of the family (**u** *_s_*) and clone within-family (**u** *_c_*) effects, aiming to account for the G×E:


(1) $$y=1\text{μ}+X_{o}\tau_{o}+Z_{s}u_{s}+Z_{c}u_{c}+Z_{b}u_{b}+e$$

where **y** ^(^ *^N^* ^× 1)^ is the vector of phenotypic observations, where *N* is the number of plots for ST data or plots by seasons for MET data; **1** ^(^ *^N^* ^× 1)^ is a vector in which all elements are unity; **μ** ^(1 × 1)^ is the intercept; **τ** *_o_* ^(^ *^o^* ^× 1)^ is the vector of fixed effects, composed of check, environment, and check by environment interaction effects (the environment and check by environment interaction effects were only used in MET data analysis), associated with matrix the design **X** *_o_* ^(^ *^N^* ^×^ *^o^* ^)^ (assuming full rank), where *o* is the number of fixed effects; **u** *_s_* ^(^ *^s^* ^× 1)^ is the vector of random effects of family associated with the design matrix **Z** *_s_* ^(^ *^N^* ^×^ *^s^* ^)^, where *s* is the number of families for ST data or families by seasons for MET data; **u** *_c_* ^(^ *^cs^* ^× 1)^ is the vector of random genotypic effects of clone within-family associated with the design matrix **Z** *_c_* ^(^ *^N^* ^×^ *^cs^* ^)^, where *c* is the number of clone within-family for ST data or clone within-family by seasons for MET data; **u** *_b_* ^(^ *^b^* ^× 1)^ is the vector of random effects of block associated with the design matrix **Z** *_b_* ^(^ *^N^* ^×^ *^b^* ^)^, where *b* is the number of blocks for ST data or blocks by seasons for MET data; and **e** ^(^ *^N^* ^× 1)^ is the vector of random errors.

We assume that the **u** *_c_*, **u** *_s_*, **u** *_b_*, and **e** vectors of random effects are mutually independent and distributed as multivariate Gaussian, with zero means and (co)variance matrices var(**u** *_c_*) = **G** *_c_*, var(**u** *_s_*) = **G** *_s_*, var(**u** *_b_*) = **G** *_b_*, and var(**e**) = **R**. The structures of these (co)variance matrices are shown in **Table 2** for ST (STMpF and STMwF) and MET (METMpF and METMwF) data analysis models, including or not the family effect, respectively. For STMwF and METMwF models, the vector of clone effects was called **u** *_c′_*. For the MET data analysis, the heterogeneity of variances of block and error effects was accommodated by the direct sum operation (⊕), whereas the heterogeneity of the variances and covariances of the G×E interaction for family and clone within-family effects was accommodated by the direct product operation (⊗). The (co)variance matrices of environments for family and clone within-family effects were modeled by an unstructured matrix with *t*(*t* + 1)/2 covariance parameters, where *t* is the number of trials.

**Table 2:** Summary of models fitted: single-trial model without family effect (STMwF), single-trial model plus family effect (STMpF), multi-environment–trial model without family effect (METMwF), and multi-environment–trial model plus family effect (METMpF).

(Co)variance matrix	Single-trial analysis		Multi-environment–trial analysis	
	STMwF	STMpF	METMwF	METMpF
**G** *_b_*	$\sigma_{b}^{2}$ **I** *_b_*	$\sigma_{b}^{2}$ **I** *_b_*	${⊕}_{j=1}^{t}\sigma_{bj}^{2}$ **I** *_bj_*	${⊕}_{j=1}^{t}\sigma_{bj}^{2}$ **I** *_bj_*
**G** *_s_*		$\sigma_{s}^{2}$ **I** *_s_*		**G** *_ts_* ⊗**I** *_s_*
**G** *_c_*	$\sigma_{c'}^{2}$ **I** *_c_* ⊗**I** *_s_*	$\sigma_{c}^{2}$ **I** *_c_* ⊗**I** *_s_*	**G** *_tc′_* ⊗**I** *_c_* ⊗**I** *_s_*	**G** *_tc_* ⊗**I** *_c_* ⊗**I** *_s_*
**R**	$\sigma^{2}$ **I** *_N_*	$\sigma^{2}$ **I** *_N_*	${⊕}_{j=1}^{t}\sigma_{j}^{2}$ **I** *_Nj_*	${⊕}_{j=1}^{t}\sigma_{j}^{2}$ **I** *_Nj_*

**G**_b_, **G**_s_, **G**_c_, and **R**: (co)variance matrices associated with the block, family, clone within-family, and error effects, respectively; **G**_ts_, **G**_tc′_, and **G**_tc_: unstructured (co)variance matrices used to accommodate the G×E interaction for the family, clone, and clone within-family effects, respectively; $\sigma_{b}^{2},\sigma_{bj}^{2},\;\sigma_{s}^{2},\;\sigma_{c'}^{2},\sigma_{c}^{2},\;\sigma^{2}$ , and $\sigma_{j}^{2}$ : variance components associated with the block, block in each trial, family, clone, clone within-family, error, and error in each trial effects, respectively; **I**_b_, **I**_bj_, **I**_s_, **I**_c_, **I**_N_ e, and **I**_Nj_: identity matrices associated with the block, block in each trial, family, clone within-family, error, and error in each trial effects, respectively; ⊗: Kronecker product operator; ⊕: direct sum operator.

The covariance parameters of models shown in **Table 2** were estimated by the residual maximum likelihood (REML) method (Patterson and Thompson, 1971) and best linear unbiased predictions (BLUP) of the random effects by Henderson’s mixed model equations (Henderson et al., 1959) through the software Echidna Mixed Models (Gilmour, 2021) version 1.61. The graphic plots and other analyses discussed in the following sections were performed using the R software (R Core Team, 2021) and R packages base and ggplot2 (Wickham, 2016).

#### 2.2.1 Single-trial analysis

From the linear mixed model for STMpF presented in **Table 2**, the vector of random total genotypic effects of clones can be predicted for ST analysis (**u** *_gST_*) by combining the vectors of family (**u** *_s_*) and clone within-family (**u** *_c_*) effects as presented in Equation (2). The (co)variance structure of the **u** *_gST_* vector is given by the composite symmetry (CS) form as shown in Equation (3):


(2) $$u_{gST}=\;(1_{c}⊗I_{s}){\;u}_{s}+u_{c}$$

(3) $$\text{var}\left(u_{gST}\right)\;=\;(\sigma_{s}^{2}J_{c}+\sigma_{s}^{2}I_{c})⊗I_{s}$$

where **1** *_c_* is a vector in which all elements are unity, **J** *_c_* is a matrix in which all elements are unity, and $\sigma_{s}^{2}$ and $\sigma_{c}^{2}$ are variance components of family and clone within-family effects, respectively.

The simple reparameterization of Equation (3), for correlation scale, allows obtaining $\rho_{S}$ correlation by Equation (4). This correlation ranges from 0 to 1 (assuming $\sigma_{s}^{2}>0$ and $\sigma_{c}^{2}>0$ ) and measures the proportion of total genetic variance due to variation among families.


(4) $$\rho_{S}=\frac{\sigma_{s}^{2}}{\sigma_{s}^{2}+\sigma_{c}^{2}}\;$$

The vector of clone effects (**u** *_c′_*) from STMwF model was also predicted. However, for STMwF, both the variance component and the BLUP of clones are confounded with family effect. Thus, the comparison between STMpF and STMwF models in terms to accuracy of total genotypic values of clones is inadequate if $\sigma_{s}^{2}$ > 0 (**Supplementary Information, Note S1**). In this context, the STMpF and STMwF models were compared using the Akaike information criterion (AIC) presented in Equation (5) (Akaike, 1974) and through the correspondence of the top 20% best clones by Czekanowski coefficient (Qiao et al., 2000) [CC, Equation (6)], as well as ranking concordance through the Spearman correlation coefficient (r*_S_*) between **u** *_c′_* and **u** *_gST_*. Furthermore, the variance components of the STMwF and STMpF models were tested using the likelihood ratio test (LRT) shown in Equation (7):


(5) AIC = −2ℓ + 2p

(6) CC = a/(a + b)

(7) $$\text{LRT }=\;-2\text{ln }\left({\text{ℓ}}_{1}/\;{\text{ℓ}}_{2}\right)$$

where ℓ is the maximum point of residual log-likelihood function, p is the number of variance parameters, a is the number of coincident clones by both selection strategies, b is the number of divergent clones by both selection strategies, ℓ_1_ is the maximum point of residual log-likelihood function from reduced model (without the effect tested), and ℓ_2_ is the maximum point of residual log-likelihood function from complete model.

Although the variance component $\sigma_{c}^{2}$ represents the average within-families genotypic variance, the BLUP of the clone within-family effect is coded to the overall mean and adjusted for the family structure, which allows for comparison of clones from different families (**Supplementary Information, Note S1**). Thus, we also compared the selection by **u** *_c_* and **u** *_gST_* vectors through the correspondence of the top 20% best clones by Czekanowski coefficient [Equation (6)], as well as ranking concordance through the Spearman correlation coefficient (r*_S_*).

To facilitate the visualization of the results, the variance components of the STMwF and STMpF models have been presented at percentage of total variation (sum of all variance components of ST analysis of the STMwF or STMpF model). To compare the efficiency of the selection strategies based on total genotypic (**u** *_gST_*) and clone within-family (**u** *_c_*) effects from STMpF, we assessed the efficiency through the accuracy ratio of respective effects.

#### 2.2.2 Multi-environment–trial analysis

The models METMwF and METMpF are extensions of the models STMwF and STMpF for MET data, respectively (**Table 2**). Similarly, to what was done for the STMpF model, the vector of total genotypic effects may also be predicted for the MET analysis using the METMpF model, combining the vectors of family (**u** *_s_*) and clone within-family (**u** *_c_*) effects as presented in Equation (8). However, unlike Equation (2), Equation (8) capitalizes the G×E. The (co)variance structure of vector **u** *_gMET_* is given by multivariate compound symmetry form as shown in Equation (9):


(8) $$u_{gMET}=\;(1_{c}⊗I_{s}⊗I_{t})us+u_{c}$$

(9) $$\text{var}\left(u_{gMET}\right)\;=\;(G_{ts}⊗J_{c}+G_{tc}⊗I_{c})⊗I_{s}$$

where **I** *_t_* is an identity matrix of trials and **G** *_ts_* and **G** *_tc_* are (co)variance matrices of trials for family and clone within-family effects.

A similar vector to the vector **u** *_gMET_* may also be predicted from the METMwF model, which was called **u** *_c′_*. However, because of the reasons highlighted in Section 2.2.1, the models METMpF and METMwF models were compared using the AIC presented in Equation (5) (Akaike, 1974) and through the correspondence of the top 20% best clones by Czekanowski’s coefficient (CC) (Qiao et al., 2000) [Equation (6)], as well as ranking concordance through the Spearman correlation coefficient (r*_S_*) based on FAI-BLUP index score (Rocha et al., 2018), between the strategies (**u** *_gMET_* vs. **u** *_c′_*).

Because of the difficulty of performing the LRT test for parameters of the unstructured matrices (**G** *_tc′_*, **G** *_ts_*, and **G** *_tc_*), two 95% confidence intervals were used for parameters of the METMwF and METMpF models. The first one, based on Chi-Square distribution (SAS Institute, 2016), used the variance components for family and clones within-family. The second is based on the Normal distribution (Meyer, 2008) for the correlations between pairs of environments for family and clone within-family effects (**Supplementary Information, Note S2**).

The genotypic correlations between the environment pairs for clone ( $\rho_{G_{c'}}$ ), family ( $\rho_{G_{s}}$ ), and clone within-family ( $\rho_{G_{c}}$ ) effects were estimated from parameters of matrices **G** *_tc′_*, v**G** *_ts_*, and **G** *_tc_*, using the expressions (10), (11), and (12):


(10) $$\rho_{G_{c'ij}}=\frac{\sigma_{c{'}_{ij}}}{\sqrt{\sigma_{c{'}_{i}}^{2}\times \sigma_{c{'}_{j}}^{2}}}$$

(11) $$\rho_{G_{sij}}=\frac{\sigma_{s_{ij}}}{\sqrt{\sigma_{s_{i}}^{2}\times \sigma_{s_{j}}^{2}}}$$

(12) $$\rho_{G_{cij}}=\frac{\sigma_{c_{ij}}}{\sqrt{\sigma_{c_{i}}^{2}\times \sigma_{c_{j}}^{2}}}$$

where $\sigma_{c{'}_{ij}}$ , $\sigma_{s_{ij}}$ , and $\sigma_{c_{ij}}$ are covariances between environments pairs *i* and *j* for clone, family, and clone within-family effects; $\sigma_{c{'}_{i}}^{2}$ and $\sigma_{c{'}_{j}}^{2}$ are variance components of environments *i* and *j* for clone effect; $\sigma_{s_{i}}^{2}$ and $\sigma_{s_{j}}^{2}$ are variance components of environments *i* and *j* for family effect; and $\sigma_{c_{i}}^{2}$ and $\sigma_{c_{j}}^{2}$ are variance components of environments *i* and *j* for clone within-family effect.

The visualization of the results and the variance components of the models METMwF and METMpF were presented in percentage of total variation for each trial (sum of all variance components for each trial of the MET analysis of the model METMwF or METMpF). To compare the effectiveness of the selection strategies based on total genotypic (**u** *_gMET_*) and clone within-family (**u** *_c_*) effects from METMpF, we assessed the effectiveness through the accuracy ratio of respective effects.

Finally, we used the FAI-BLUP index (Rocha et al., 2018), to rank the clones based on predicted BLUPs of MET data for both strategies (**u** *_gMET_* vs. **u** *_c_*). The two selection strategies have been compared as described before. Conducted the exploratory factor analysis, with Factor Analysis (FA) and Principal Component Analysis (PCA). PCA was used to extract the factorial loads from the genetic correlation matrix, obtained by the predicted values (BLUPs). The analysis used is varimax criterion (Kaiser, 1958) for the analytic rotation and the calculation of the factor scores of the weighted least squares method (Bartlett, 1938). Thus, PCA and FA were performed on the set of BLUP mean for six variables from population 2, three seasons (POP2HHS, POP2MHS, and POP2WHS), and two traits (TTY and SG) of each vector **u** *_c_*, **u** *_c’_*, and **u** *_gMET_*. They were estimated from multi-environment–trial model without the family effect (METMwF) and multi-environment–trial model plus the family effect (METMpF).

#### 2.2.3 Accuracy of family, clone within-family, total genotypic effects, and relative efficiency

The accuracy of family ( $r_{\hat{s}s}$ ), clone within-family ( $r_{\hat{c}c}$ ), total genotypic effects ( $r_{\hat{g}g}$ ), and relative efficiency (RE) were obtained by expressions (13), (14), (15), and (16) for both ST and MET data analysis:


(13) $${\text{r}}_{\hat{s}s}=\sqrt{1-\frac{\upsilon_{s}}{\sigma_{s}^{2}}}\;$$

(14) $${\text{r}}_{\hat{c}c}=\sqrt{1-\frac{\upsilon_{c}}{\sigma_{c}^{2}}}$$

(15) $${\text{r}}_{\hat{g}g}=\sqrt{1-\frac{\upsilon_{g}}{\sigma_{s}^{2}+\sigma_{c}^{2}}}$$

(16) $$RE=\frac{{\text{r}}_{\hat{g}g}}{{\text{r}}_{\hat{c}c}}$$

where $\upsilon_{s}$ , $\upsilon_{c}$ , and $\upsilon_{g}$ are the average prediction error variance of family, clone within-family, and total genotypic effects, respectively. The RE was used to measure the difference between the proposed models to ST and MET data.

## 3 Results

### 3.1 Comparison of the STMwF and STMpF models

The inclusion of the family effect improved the goodness of fit of the models in all trials, on which the STMpF model showed lower AIC than the STMwF, and the only exception was for TTY from POP3(WHS) (**Table 3**). In addition, the inclusion of family effect also increased the log-likelihood (ℓ) in all trials for both traits. Only for the TTY trait in the POP3(WHS) trial did this increment does not exceed 1.92 units (critical point for the detection of significant $\sigma_{s}^{2}$ effect) (**Table 3**). These results are reinforced by the results of the LRT test (**Table 4**) and indicate the presence of genetic variability between families in most trials.

**Table 3:** Log-likelihood residual (ℓ) and Akaike information criterion (AIC) for single-trial model without family effect (STMwF) and single-trial model plus family effect (STMpF), for all trials and traits.

Trait^‡^	Model	Trial^†^	ℓ	AIC
TTY	STMwF	POP1(WHS)	−1,826.81	3,659.63
		POP2(WHS)	−1,459.44	2,924.89
		POP2(MHS)	−1,584.44	3,174.88
		POP2(HHS)	−1,191.74	2,389.47
		POP3(WHS)	−1,031.07	2,068.14
		POP3(HHS)	−1,155.63	2,317.27
		POP1(WHS)	−1,820.36	3,648.72
	STMpF	POP2(WHS)	−1,450.92	2,909.84
		POP2(MHS)	−1,581.43	3,170.85
		POP2(HHS)	−1,189.63	2,387.26
		POP3(WHS)	−1,030.43	2,068.85
		POP3(HHS)	−1,149.94	2,307.87
SG	STMwF	POP1(WHS)	2,587.78	−5,169.57
		POP2(WHS)	2,200.75	−4,395.51
		POP2(MHS)	2,262.34	−4,518.68
		POP2(HHS)	1,822.53	−3,639.06
		POP3(WHS)	1,278.39	−2,550.77
		POP3(HHS)	1,591.35	−3,176.70
		POP1(WHS)	2,613.73	−5,219.45
	STMpF	POP2(WHS)	2,209.84	−4,411.68
		POP2(MHS)	2,276.28	−4,544.56
		POP2(HHS)	1,833.07	−3,658.15
		POP3(WHS)	1,281.73	−2,555.45
		POP3(HHS)	1,594.28	−3,180.55

^‡^Total tuber yield (TTY; Mg ha^−1^) and specific gravity (SG).

**^†^** The trial identification: POP1(WHS), POP2(WHS), POP2(MHS), POP2(HHS), POP3(WHS), and POP3(HHS), where the codes POP1, POP2, and POP3 identify the different clonal populations and the codes WHS, MHS, and HHS identify three different seasons, varying in function of stress level: without heat stress (WHS) moderate heat stress (MHS), and high heat stress (HHS).

**Table 4:** Contribution (%) of the variances of block, clone (C′), family (F), clone within-family (C), and residuals (Res.) for the phenotypic variance of the traits total tuber yield (TTY; Mg ha^−1^) and specific gravity (SG) estimated from single-trial model without family effect (STMwF) and single-trial model plus family effect (STMpF) in different seasons.

Trait	Trial^†^	STMwF			STMpF				
		Block	Clone (C′)	Res.	Block	Family (F)	Clone (C)	Res.	$\rho_{S}$ ^‡^
TTY	POP1(WHS)	2.67^ns^	40.97^**^	56.35	2.05^ns^	7.18^**^	34.42^**^	56.35	0.17
	POP2(WHS)	7.88^**^	47.78^**^	44.34	5.50^**^	11.56^**^	35.56^*^	47.38	0.26
	POP2(MHS)	3.88^**^	37.74^*^	58.38	3.77^**^	4.43^**^	32.62^*^	59.18	0.12
	POP2(HHS)	0.22^ns^	69.55^**^	30.23	0.19^ns^	4.38^*^	65.25^**^	30.18	0.06
	POP3(WHS)	7.13^**^	77.27^**^	15.61	7.26^**^	2.32^ns^	74.90^**^	15.52	0.03
	POP3(HHS)	1.09^ns^	68.44^**^	30.47	1.61^ns^	8.76^**^	59.62^**^	30.01	0.13
SG	POP1(WHS)	9.83^**^	69.87^**^	20.30	7.69^**^	18.56^**^	53.15^**^	20.60	0.26
	POP2(WHS)	12.76^**^	35.00^*^	52.24	5.03^ns^	15.11^**^	28.43^*^	51.43	0.35
	POP2(MHS)	2.26^*^	43.40^*^	54.34	2.10^ns^	13.60^**^	29.94^*^	54.36	0.31
	POP2(HHS)	3.91^**^	56.78^**^	39.31	3.57^*^	12.97^**^	44.10^**^	39.36	0.23
	POP3(WHS)	3.29^*^	57.63^**^	39.09	3.42^*^	6.75^**^	49.88^**^	39.96	0.12
	POP3(HHS)	8.01^**^	58.58^**^	33.41	7.97^**^	5.04^**^	52.42^**^	34.56	0.09

**^†^** The trial identification: POP1(WHS), POP2(WHS), POP2(MHS), POP2(HHS), POP3(WHS), and POP3(HHS), where the codes POP1, POP2, and POP3 identify the different clonal populations and the codes WHS, MHS, and HHS identify three different seasons, varying in function of stress level: without heat stress (WHS) moderate heat stress (MHS), and high heat stress (HHS).

**^‡^** $\rho_{S}$ correlation: measures the proportion of total genetic variance due to variation among families.

Significance by the likelihood-ratio test (LRT): p-value< 0.01 “**” and 0.05 “*” and p-value > 0.05 not significant “^ns^”.

Clone ( $\sigma_{c'}^{2}$ , STMwF) and clone within-family ( $\sigma_{c}^{2}$ , STMpF) variances were significant in all trials for both traits (**Table 4**; **Supplementary Material, Table S2**), revealing the existence of genetic variability among clones. The contribution of clone within-family variance (C) to the total phenotypic variance was always lower than the clone effect (C′) (**Table 4**). However, the magnitude of the difference between C′ and C was directly proportional to the contribution of family variance to the total genetic variation ( $\rho_{S}$ ). The amplitude of $\;\rho_{S}$ for the SG trait (0.09 and 0.35) exceeded the amplitude for the TTY trait (0.03 and 0.26) (**Table 4**).

**Figure 2:**
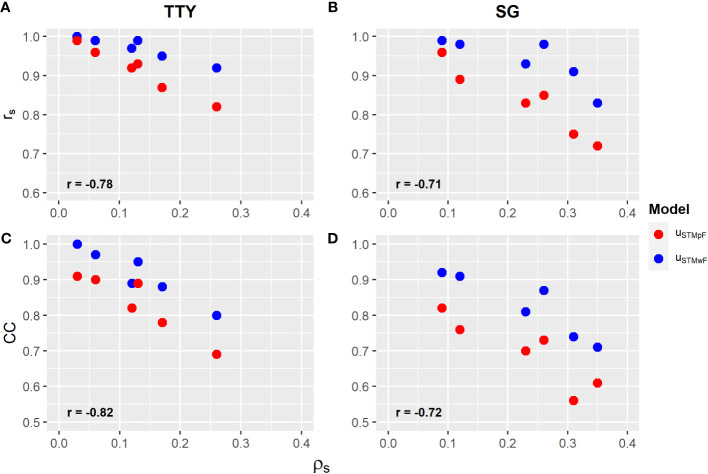
**(A, B)** Spearman correlation coefficient (r*_S_*), and **(C, D)** Czekanowski coefficient (CC) (**Supplementary Material, Tables S3, S4**) between the proportion of total genetic variance of families ( $\rho_{S}$ ) (**Table 4**) for the vector of clone effects of models from the single trial without family (u_STMwF_) and plus family effect (u_STMpF_) to the total tuber yield (TTY; Mg ha^−1^) and specific gravity (SG). Labels in bold are correlations between selective efficiency and $\rho_{S}$ .

CC and Spearman’s correlation coefficient (r*_S_*) were used as comparison criterion for both selection strategies tested (**u** *_c′_* vs. **u** *_gST_* and **u** *_c_* vs. **u** *_gST_*). It was observed that, regardless of the selection strategy and trait, both coefficients showed an inverse relationship with $\rho_{S}$ , suggesting that an increase in genetic variability among families reduces the similarity of the **u** *_c′_* and **u** *_c_* vectors with the **u** *_gST_* vector in the ranking of clones. Furthermore, the magnitude of the correlations of the CC and r*_S_* coefficients with $\rho_{S}$ was higher for the TTY (−0.82 and −0.78) compared with that for the SG (−0.72 and −0.71) (**Figure 2**).

In general, both CC and r*_S_* showed lower magnitude for the second selection strategy with family effect (**u** *_c_* vs. **u** *_gST_*), which suggests a greater agreement of the **u** *_c′_* vector without family effect with **u** *_gST_* in the clone ranking (**Figure 2**; **Supplementary Material**, **Tables S4, S5**). Furthermore, independent of the selection strategy, an increment in CC and r*_S_* can be observed with increasing heat stress for both traits in POP2 and POP3, except for the TTY in population POP3 (**Supplementary Material, Tables S4, S5**).

### 3.2 Relative efficiency of selection based on total genotypic effect of clone for ST analysis

**Figure 3:**
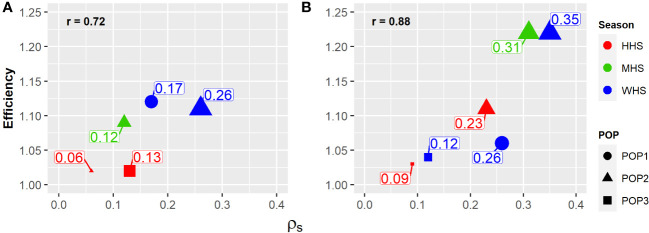
Family effect response ( $\rho_{S}$ ) (size) on the selective accuracy of potato clones (relative efficiency) in the function of heat stress level (color): without heat stress (WHS), moderate heat stress (MHS), and high heat stress (HHS), in three populations evaluated (shape): POP1, POP2, and POP3. **(A)** Total tuber yield (TTY; Mg ha^−1^) and **(B)** specific gravity (SG).

Regardless of the trait, selection based on the vector of total genotypic effects of clone (**u** *_gST_*) was found to be greater than the selection based on the vector of clone effects within family (**u** *_c_*) (**Table 5**). The efficiency was directly proportional to $\rho_{S}$ , showing that the increment in genetic variability among families increases the selective efficiency of clones. The correlation between efficiency and $\rho_{S}\;$ was higher for SG (0.88) when compared with that for TTY (0.72) (**Figure 3**).

**Table 5:** Accuracy of the family ( ${\text{r}}_{\hat{s}s}$ ), clone within-family (.), and total genotypic ( ${\text{r}}_{\hat{g}g}$ ) effects for traits total tuber yield (TTY; Mg ha^−1^) and specific gravity (SG) estimated from single-trial model plus family effect (STMpF) in different seasons.

Trait	Trial^†^	${\text{r}}_{\hat{s}s}$	${\text{r}}_{\hat{c}c}$	${\text{r}}_{\hat{g}g}$	Efficiency^‡^
TTY	POP1(WHS)	0.74	0.60	0.67	1.12
	POP2(WHS)	0.76	0.64	0.71	1.11
	POP2(MHS)	0.63	0.58	0.63	1.09
	POP2(HHS)	0.60	0.81	0.83	1.02
	POP3(WHS)		0.90**^§^**		
	POP3(HHS)	0.81	0.81	0.84	1.02
	Average	0.71	0.69	0.74	1.07
SG	POP1(WHS)	0.87	0.82	0.87	1.06
	POP2(WHS)	0.80	0.58	0.71	1.22
	POP2(MHS)	0.82	0.58	0.71	1.22
	POP2(HHS)	0.79	0.70	0.78	1.11
	POP3(WHS)	0.77	0.75	0.78	1.04
	POP3(HHS)	0.73	0.78	0.80	1.03
	Average	0.80	0.70	0.78	1.11

**^†^** The trial identification: POP1(WHS), POP2(WHS), POP2(MHS), POP2(HHS), POP3(WHS), and POP3(HHS), where the codes POP1, POP2, and POP3 identify the different clonal populations and the codes WHS, MHS, and HHS identify three different seasons, varying in function of stress level: without heat stress (WHS) moderate heat stress (MHS), and high heat stress (HHS).

**^‡^** Relative efficiency: ${\text{r}}_{\hat{g}g}/{\text{r}}_{\hat{c}c}$ ratio.

^§^Not included in estimate of average accuracy.

On average, the accuracies ${\text{r}}_{\hat{s}s}$ , ${\text{r}}_{\hat{c}c}$ , and ${\text{r}}_{\hat{g}g}$ were higher for SG (0.80, 0.70, and 0.78, respectively) compared with that for TTY (0.71, 0.69 and 0.74), respectively. The difference between the accuracies ${\text{r}}_{\hat{g}g}$ and ${\text{r}}_{\hat{c}c}$ was higher for SG (0.78 and 0.70) than that for TTY (0.74 and 0.69), which resulted in higher average selective efficiency for the SG (11%) compared with that for TTY (7%) (**Table 5**).

### 3.3 Comparison of the METMwF and METMpF models

Variance component associated with family effect in the trial POP3(WHS) was not significant by the LRT test for TTY (**Table 4**) and, thus, was not included in the MET analysis. The family effect (METMpF) did not improve the goodness of fit compared with the METMwF model for POP3 (**Table S5**); thus, the MET analysis results were presented only for POP2 (**Table 6**).

**Table 6:** Contribution (%) of the variances of blocks, clones (C′), families (F), clone within-family (C), and residuals (Res.) for the phenotypic variance of the traits total tuber yield (TTY; Mg ha^−1^) and specific gravity (SG) estimated from multi-environment–trial model without family effect (METMwF) and multi-environment–trial model plus family effect (METMpF) in different seasons.

Trait	Trial^†^	METMwF			METMpF				
		Block	Clone (C′)	Res.	Block	Family (F)	Clone (C)	Res.	$\rho_{S}$ ^‡^
TTY	POP2(WHS)	8.75^+^	48.17^+^	43.09^+^	5.80^+^	11.26^+^	36.45^+^	46.48^+^	0.24
	POP2(MHS)	3.22^+^	38.34^+^	58.44^+^	3.00^+^	4.81^+^	33.08^+^	59.11^+^	0.13
	POP2(HHS)	0.00^#^	70.02^+^	29.98^+^	0.00^#^	4.38^+^	65.70^+^	29.92^+^	0.06
SG	POP2(WHS)	12.63^+^	33.33^+^	54.04^+^	6.37^+^	13.50^+^	28.03^+^	52.10^+^	0.33
	POP2(MHS)	1.53^+^	43.06^+^	55.41^+^	1.52^+^	13.79^+^	29.70^+^	54.99^+^	0.32
	POP2(HHS)	3.81^+^	56.31^+^	39.88^+^	3.47^+^	12.71^+^	44.13^+^	39.69^+^	0.22

**^†^** The trial identification: POP2WHS, POP2MHS, and POP2HHS, where the code POP2 identify the clonal population and codes WHS, MHS, and HHS identify three different seasons, varying in function of stress level: without heat stress WHS, moderate heat stress MHS, and high heat stress HHS.

**^‡^** ρS correlation: measures the proportion of total genetic variance due to variation among families.

**^+^** Variance component does not intercept the zero by 95% Chi-Squared confidence intervals.

**^#^** Variance component intercept the zero by 95% Chi-Squared confidence intervals.

The variance estimates associated with the effects of clone ( $\sigma_{c'}^{2}$ , METMwF), family ( $\sigma_{s}^{2}$ , METMpF), and clone within-family ( $\sigma_{c}^{2}$ , METMpF), as well as their respective contributions to the phenotypic variance, were similar to those obtained in the ST analyses in all trials and traits. The variance components associated with clone, family, and clone within-family were higher than zero in all scenarios (**Table S6**), confirming the results found in the ST analyses (**Tables 4**, **6**; **Supplementary Material**, **Tables S6, S7**). It is worth noting that the values of $\rho_{S}\;$ were also like those obtained in the ST analyses (**Tables 4**, **6**).

Overall, variation was observed in the estimates of variance components $\sigma_{c'}^{2}$ , $\sigma_{s}^{2}$ , and $\sigma_{c}^{2}$ across the different seasons for both traits, indicating that populations that have G×E can be attributed to the interaction of a simple nature (**Supplementary Material, Table S8**). Genetic correlation between seasons for the effects of clone, family, and clone within-family was positive in all scenarios for SG, with values higher than 0.50 in most cases. This suggests a low contribution of complex type G×E for this trait. In contrast, most genetic correlation estimates for TTY were lower than 0.50, suggesting a greater contribution of the complex G×E (**Supplementary Material, Tables S6, S7**).

Regardless of the model and trait, the variance component estimates associated the effects of clone ( $\sigma_{c'}^{2}$ ) and clone within-family ( $\sigma_{c}^{2}$ ) were always higher in the higher heat stress season (HHS) when compared with that in the no heat stress season (WHS), indicating an increase in genetic variability under extreme heat stress (**Supplementary Material, Table S1**). The variance component associated with family effect ( $\sigma_{s}^{2}$ ) showed a behavior inversely proportional to the increase of heat stress for the TTY trait, reducing about 50% with the increment of heat stress [11.32 (WHS), 7.32 (MHS), and 3.64 (HHS)] (**Supplementary Material, Table S3**). Furthermore, regardless of the model adopted, we recorded reductions of 42% and 3% in the average of the clones in the POP2 population for the traits TTY and SG under HHS, respectively (**Supplementary Material, Table S3**). It is worth noting that, considering the period from the beginning of tuberization (about 30 days after planting) until harvest, the average daily temperature exceeded 20°C on 64% of the days in the MHS season and 88% of the days in the HHS season (88%) (**Figure 1**).

The exploratory factor analysis showed mean communality of 0.91, 0.91, and 0.87 for the respective vectors **u** *_c′_*, **u** *_c_*, and **u** *_gMET_*, respectively, indicating that the three factors were sufficient to explain more than 87% of the relationship between seasons. Regardless of the effect, Factor 1 represented the three seasons for SG, Factor 2 represented the WHS and MHS seasons for TTY, and Factor 3 represented only the HHS season (**Supplementary Material, Table S8**).

After obtaining the FAI-BLUP index scores, which included both traits and all seasons, the Czarnowski’s coefficients (CC) and Spearman’s correlation (r*_S_*) were utilized to compare the two selection strategies tested (**u** *_c′_* vs. **u** *_gMET_* and **u** *_c_* vs. **u** *_gMET_*). Similarly, to what was observed for the ST analysis, both CC and r*_S_* showed lower magnitude for the second selection strategy (**u** *_c_* vs. **u** *_gMET_*), which suggests closer concordance of the vector **u** *_c′_* with **u** *_gMET_* in the ranking of the clones (**Table 7**).

**Table 7:** Czekanowski’s coefficient (CC) and Spearman’s correlation (r*_S_*) between the FAI-BLUP index score vectors of different strategies (**u** *_c′_* vs. **u** *_gMET_* and **u** *_c_* vs. **u** *_gMET_*), for traits total tuber yield (TTY; Mg ha^−1^) and specific gravity (SG).

Strategies^†^	CC	r*_S_*
**u** *_c′_* vs. **u** *_gMET_*	0.94	0.98
**u** *_c_* vs. **u** *_gMET_*	0.82	0.92

^†^ **u**_c′_ is the vector of clone effects from multi-environment–trial model without family effect (METMwF), **u**_c_ is the vector of clone’s effects from multi-environment–trial model with family effect (METMpF), and **u**_gMET_ is the vector of total genotypic effects of clones from METMpF.

### 3.4 Relative efficiency of selection based on the total genotypic effect of clone for MET analysis

Similar to the ST results, independent of trait or season, the selection based on the vector of genotypic effect of clones in the MET (**u** *_gMET_*) was higher than the selection based on the vector of clone within-family in the MET (**u** *_c_*) (**Table 8**). It is worth noting that, because of the relationship between the season’s pairs, the accuracy associated with the within-family clone effect was increased in all seasons for both traits, although the accuracy associated the family effect was maintained or was increased (**Table 8**).

**Table 8:** Accuracy of family ( ${\text{r}}_{\hat{s}s}$ ), clone within-family ( ${\text{r}}_{\hat{c}c}$ ), and total genotypic ( ${\text{r}}_{\hat{g}g}$ ) effects for traits total tuber yield (TTY; Mg ha^−1^) and specific gravity (SG) estimated from multi-environment–trial model plus family effect (METMpF) in different seasons.

Trait	Trial^†^	${\text{r}}_{\hat{s}s}$	${\text{r}}_{\hat{c}c}$	${\text{r}}_{\hat{g}g}$	Efficiency^‡^
TTY	POP2(WHS)	0.76	0.67	0.73	1.09
	POP2(MHS)	0.68	0.65	0.69	1.06
	POP2(HHS)	0.65	0.82	0.84	1.02
	Average	0.70	0.71	0.75	1.06
SG	POP2(WHS)	0.80	0.67	0.75	1.12
	POP2(MHS)	0.84	0.69	0.78	1.13
	POP2(HHS)	0.82	0.73	0.80	1.10
	Average	0.82	0.70	0.78	1.12

**^†^** The trial identification: POP2(WHS), POP2(MHS), POP2(HHS), where the code POP2 identify the clonal population and codes WHS, MHS, and HHS identify three different seasons, varying in function of stress level: without heat stress (WHS) moderate heat stress (MHS), and high heat stress (HHS).

**^‡^** Relative efficiency: ${\text{r}}_{\hat{g}g}/{\text{r}}_{\hat{c}c}$ ratio.

For the TTY trait, efficiency estimates were progressively reduced with increasing heat stress levels [1.09 (WHS), 1.06 (MHS), and 1.02 (HHS)], although, for the SG trait, efficiencies were similar in all seasons. On average, the accuracies ${\text{r}}_{\hat{s}s}$ , ${\text{r}}_{\hat{c}c}$ , and ${\text{r}}_{\hat{g}g}$ were higher for the SG trait (0.82, 0.70, and 0.78) in comparison with the TTY trait (0.70, 0.71, and 0.75), respectively. Furthermore, the magnitude of the difference between the accuracies ${\text{r}}_{\hat{g}g}$ and ${\text{r}}_{\hat{c}c}$ was higher for the SG trait (0.78 and 0.70) detriment of TTY (0.75 and 0.71), which resulted in higher average selective efficiency for the SG trait (12%) when compared to the TTY trait (6%) (**Table 8**).

## 4 Discussion

### 4.1 ST analyses

The results presented here indicate that adjusting for the structure created by family effects can improve the estimation of genetic values. However, it is important to highlight that phenotyping individual data in the early stages of the process may not be compatible with certain breeding pipelines. For example, in certain programs, the first-year evaluation takes place in the field, with mass selection, and using single-hill trials (one seed potato). In this case, the extra phenotyping and modeling of the data would incur additional resources, and the feasibility of such change needs to be evaluated on a case-by-case basis. One technology that potentially can be used to facilitate data collection in the early stages of the program would be the use of aerial images for phenotyping tubers that are dug out of the ground but left in the field (Matias et al., 2020). In contrast, in cases where the early stages are already established with larger plots and randomization of clones, the inclusion of the family effect in the quantitative genetics model would come at no additional effort in data collection.

The genetic progress obtained through conventional potato breeding is slow due to the complexity of the tetrassomic inheritance, the elevated level of heterozygosity of the genitors, and the large number of traits to be assessed (~40) (Haynes et al., 2012; Slater et al., 2014b; Gopal, 2015; Bradshaw, 2017). Potato-breeding programs generate thousands of seedlings annually to overcome these challenges (Stich and Van Inghelandt, 2018). Furthermore, initial stage of potato breeding has low availability of seeds because of the slow tuber multiplication rates, which restricts the use of repetitions and plot size (Paget et al., 2017). These factors contribute to lower experimental precision and selection accuracy.

Typically, the clones being evaluated come from various families, which are often created through biparental crossings. Therefore, the selection process involves making comparisons not only between clones from the same family but also between those from different families. Thus, the effect of clones, and its variance component, are confounded with the family effect. To address this problem and achieve higher selection accuracy, is the use of a nested model, on which the effect of clones is nested within the family effect (Family/Clone = Family + Family + Clone). According to Piepho et al. (2008), models with this structure can be advantageous as they implicitly account for the genetic relatedness of clones. It is important to highlight that including the family effect enhances the model performance due to a better representation of the families, once the clones within a family work as repetitions.

Resende et al. (2016) conducted a study on the effectiveness of the nested structure in a dry bean breeding program using progenies from various populations. They emphasized the significance of the population effect, as the use of the nested model resulted in 20 times more repetitions for each population compared to the progenies. Resulted from an increase in the selective efficiency of progenies. Some papers that considered the effect of populations are reported on bean (Resende et al., 2016; Paula et al., 2020) and soybean (Pereira et al., 2017; Volpato et al., 2018) crops. Thus, the nested structure allows obtaining a more accurate BLUP of the family, which enables a more reliable selection of the best clones. In addition, it allows for the selection of the best families, which enables the evaluation of a greater number of clones per family and increased plot size, thereby increasing the probability of obtaining superior clones.

The utilization of a nested structure presents a significant advantage in providing precise estimations of genetic parameters. This is because the variance components within and between families are not confounded, which can happen in models that do not take family effects into account (**Supplementary Material, Note S1**). In the former, the heritability of clones is overestimated due to the variance component of family, whereas, in the nested models, the family structure gives a better estimate of the clone effect (**Supplementary Material, Note S1**). The effects of clone and clone within-family are only equivalent when the variance component of families is zero or close to zero. Pereira et al. (2017) indicates that incorporating the population effect into the estimation of genetic and non-genetic components in soybean breeding data offers a more accurate and realistic methodology.

Higher accuracy was observed using the vector of total genotypic effects (**u** *_gST_*), obtained by combining the vectors of family effects (**u** *_s_*) and clone within-family (**u** *_c_*), and it was directly proportional to the contribution of the variance component associated with family in the total genetic variation ( $\rho_{S}$ ). Resende et al. (2016) in a simulation study showed that the selection accuracy increases in function of the increased contribution of the between-population variance component to the total genetic variation. Therefore, although, in potato breeding, the genetic variance between families is lower than the genetic variance within families, the inclusion of the effect of family can increase the selection accuracy because the heritability of families has been, in general, higher than the heritability of clone within-family (Diniz et al., 2006; Melo et al., 2011). Greater average RE recorded for the SG trait is associated with the higher mean accuracy for the effect of families (0.80) and the higher $\rho_{S}$ mean (0.23).

The utilization of the family effect in potato breeding, which models the nested effect of clones within the family, has been shown to be advantageous. This methodology has led to an improvement in the accuracy of predicting the genotypic values of clones and has achieved a greater degree of RE for the two traits that were studied. The increase in accuracy achieved by the nested model does not reflect increased costs in cases where the data are already recorded. We expect that animal modeling properly accounting for additive and dominance effects would also result in more efficient results. However, the results showed that family effect inclusion is a simpler approach, mainly in cases where the mating design is not complete.

### 4.2 MET analyses

Understanding and effectively managing the G×E interaction is essential for achieving long-term improvements in plant breeding programs because the success of a new cultivar depends on its improved performance on different traits (e.g., TTY and SG) while also presenting good adaptability and stability. The G×E assumes a particular importance for potato breeding, mainly under tropical conditions, because heat stress limits yield and quality in the hottest periods of the year (Fleisher et al., 2017; Fernandes Filho et al., 2021; Patiño-Torres et al., 2021).

Potato crop generally presents better performance in regions with temperate climate, with average temperatures between 5°C and 21°C (Haverkort and Verhagen, 2008). With temperature exceeding 21°C, heat stress significantly reduces tuber yield and quality, due to a series of physiological changes, including an increase in tuber disorders (e.g., internal heat necrosis, knobs, and tuber chain), reduced plant growth, increased respiration rate, greater allocation of dry biomass to leaves at the expense of the tubers, and the reduction in photosynthetic pigments (Lambert et al., 2006; Haverkort and Verhagen, 2008; Hancock et al., 2014; Rykaczewska, 2015; Patiño-Torres et al., 2021). Thus, the selection of heat-tolerant clones is vital to increase potato tuber yield and quality.

Brazil potato season is carried out in three distinct seasons: dry (January to March), winter (April to July), and water (August to December) seasons. Temperatures above the critical threshold, 21°C, are commonly recorded in the dry and water seasons (Andrade et al., 2021). Fernandes Filho et al. (2021) classified these seasons according to their level of heat stress: MHS, WHS, and HHS. One of the major limiting factors for Brazilian potato yield is the use of cultivars that are poorly adapted for tropical conditions and hot temperatures.

The Universidade Federal de Lavras’s potato-breeding program has worked intensively developing heat-tolerant clones (Benites and Pinto, 2011; Figueiredo et al., 2015; Fernandes Filho et al., 2021; Patiño-Torres et al., 2021). To determine which clones, have high heat tolerance, they are tested under varying levels of heat stress. Only clones that perform well under mild and high temperatures are selected (Benites and Pinto, 2011; Rykaczewska, 2015; Fernandes Filho et al., 2021; Patiño-Torres et al., 2021). Furthermore, Andrade et al. (2021) reported that environments with a higher level of heat stress showed a greater capacity to differentiate clones according to their performance.

The strategy mentioned above for selecting heat-tolerant clones requires clones to be evaluated in two or more contrasting environments regarding heat stress. According to Smith et al. (2001), to better evaluate G×E, it is more realistic to use models that consider variance heterogeneity and genetic covariances when analyzing MET data. Cullis et al. (1998) and Smith et al. (2001) reported a significant reduction in the log-likelihood REML when homogenous variance was assumed for the G×E. In multiplicative models for MET data, the interaction of simple nature is accounted by the heterogeneity of genetic variances along the environments, whereas the complex interaction is accounted by the heterogeneity of genetic correlations between pairs of environments (Crossa et al., 2004; Eeuwijk et al., 2016). According to Eeuwijk et al. (2016), when the genetic correlations between two environments are high, it means that there is a smaller percentage of complex interaction involved.

MET data analysis can be carry out in one or two stages (Cullis et al., 1998; Smith et al., 2001; Smith et al., 2015; Gogel et al., 2018). Although the two-stage analysis is often employed, under unbalance (varying number of genotypes between environments or varying number of repetitions between and within environments), a common condition in the early stage of potato-breeding programs, the MET data analysis in one stage is more efficient (Cullis et al., 1998; Smith et al., 2001; Paget et al., 2017; Gogel et al., 2018).

Although the analysis of MET data is advantageous because of the advantage of the interrelationship between environments, it is increasing selective accuracy and allows a better interpretation of the G×E interaction (Smith et al., 2001; Kelly et al., 2007). The family effect, naturally present in the initial stage of potato-breeding programs, has also been neglected in this type of analysis (Fernandes Filho et al., 2021). However, one can combine the advantages cited above with those described in Section 4.1. Making MET analysis a powerful tool for clone selection in the early stages of potato-breeding programs, especially under tropical conditions, it is opportune to highlight that the recovery of inter-environmental information was more pronounced for the effect of clones within-family because of the higher magnitude of genetic correlations between seasons. This corroborates with the lower magnitude of the selective efficiency in MET analysis comparison to ST analysis. Selective efficiency for the TTY trait progressively reduced with increasing heat stress levels, whereas the selective efficiency for the SG trait was maintained practically constant. These results ratify the importance of environments with higher heat stress levels to discriminate potato clones for the TTY trait, as well as the greater contribution of the family effect to the selection of superior clones for the SG trait.

The unstructured model can be used in cases when only a few trials are included, due to the smaller number of parameters that need to be estimated, avoiding the use of factor analysis (Smith et al., 2001; Melo et al., 2020; Fernandes Filho et al., 2021). However, the use of the unstructured model implies the prediction of genotypic effects for each trial (Kelly et al., 2007; Eeuwijk et al., 2016; Melo et al., 2020; Fernandes Filho et al., 2021). Hence, it is desirable to use a selection index to capture the G×E interaction and to combine MET analyses realized for multiple traits (Kelly et al., 2007; Melo et al., 2020; Fernandes Filho et al., 2021).

There are many options for selection indexes (Mendes et al., 2009; Rocha et al., 2018; Yan and Frégeau-Reid, 2018; Melo et al., 2020). Among the different indexes, the FAI-BLUP index stands out, because it can incorporate data from various environments and traits without the need for weights. It also avoids issues with multicollinearity and makes it easier to understand the G×E interaction through exploratory factor analysis (Rocha et al., 2018; Oliveira et al., 2019). In the present study, factor analysis showed a superior performance in summarizing the relationships among environments (WHS, MHS, and HHS) and traits (TTY and SG) for each effect (**u** *_c′_*, **u** *_c_*, and **u** *_gMET_*). In addition, the factor analysis showed that the SG trait had a lower level of interaction because all seasons were grouped under Factor 1. On the other hand, the TTY trait had a higher level of interaction as the WHS and HHS seasons were grouped under distinct factors. Thus, including the effect of families in the MET analysis is a helpful strategy to increase the accuracy of superior clone selection.

Finally, the inclusion of the family effect increased the selective efficiency of clones in ST and MET selection on schemes through an increment in the accuracy of the total genotypic value. On average, the selective efficiency of clones was 11% and 7% for ST and 12% and 6% in MET for the traits SG and TTY, respectively. An expressive reduction of the family effect under heat stress for TTY and of lower magnitude for SG was observed.

Thus, the results of the present work suggest that the inclusion of the family effect in clone selection models, in the initial stage of potato-breeding programs, is desirable because it contributes to increasing the selective efficiency of clones without generating additional costs, especially for the SG trait.

## Data availability statement

The original contributions presented in the study are included in the article/**Supplementary Material.** Further inquiries can be directed to the corresponding authors.

## Author contributions

VM: Conceptualization, Data curation, Formal Analysis, Investigation, Methodology, Project administration, Writing – original draft, Writing – review & editing. MA: Supervision, Writing – review & editing. LP: Data curation, Writing – review & editing. LM: Data curation, Writing – review & editing. CF: Data curation, Writing – review & editing. MG: Data curation, Writing – review & editing. JN: Supervision, Writing – review & editing. LJ: Supervision, Writing – review & editing. LZ: Supervision, Writing – review & editing. MR: Supervision, Writing – review & editing. PC: Supervision, Writing – review & editing. TM: Conceptualization, Formal Analysis, Investigation, Methodology, Project administration, Supervision, Validation, Writing – original draft, Writing – review & editing.
