# Supplementary material for: Evaluating the impact of modeling the family effect for clonal selection in potato-breeding programs
Source: Front Plant Sci. 2023 Oct 30;14:1253706. doi: 10.3389/fpls.2023.1253706 (PMC10642306; doi:10.3389/fpls.2023.1253706)
Supplement: Supplementary file 1 [file DataSheet_1.pdf]

Supplementary Material

Contents

1. **Supplementary note**

   Note S1 (pages 2 and 3)

   Note S2 (pages 4 and 5)

2. **Supplementary tables**

   Table S1 and Table S2 (page 5)

   Table S3 and Table S4 (page 6)

   Table S5 and Table S6 (page 7)

   Table S7 (page 8)

   Table S8 (page 9)

3. **Supplementary references**

1. Supplementary notes

Note S1. Relationship between models STMpF and STMwF

Using the notation given by Piepho et al. (2008), the nested structure **Family/Clone** resolves as **Family + Family·Clone**, where the dot indicates a crossed effect. Considering a randomized block trial (RBD) with b blocks, s families, and c within-family clones, the ANOVA table, including the adapted mean squares expectations of Resende et al. (2016), is equal to:

Table 1-Note S1. Overview of ANOVA table for the nested structure of Family/Clone.

Sources of Variation	Degree of Freedom	Mean Square	E(Mean Square)
Block	b-1	MS_b	
Family	s-1	MS_s	$\sigma^2 + b\sigma_c^2 + bs\sigma_s^2$
Family·Clone	$s(c$-1)	MS_c	$\sigma^2 + b\sigma_c^2$
Error	(b-1)(sc - 1)	MS_e	σ^2

In the case where the family effect is not included in the analysis, the ANOVA table is equal to:

Table 2-Note S1. Overview of ANOVA table without family effect.

Sources of Variation	Degrees of Freedom	Mean Square	E(Mean Square)
Block	b-1	MS_b	
Family·Clone	(sc-1)	$MS_{c'}$	$\sigma^2 + b\sigma_{c'}^2$
Error	(b-1)(sc - 1)	MS_e	σ^2

Comparing the two analyses, it is possible to verify that the clone mean square of the analysis without family effect ($MS_{c'}$, Table 2-Note S1) is equal to the weighted mean of the mean squares of the family (MS_s) and within-family clone (MS_c) of the analysis including the family effect, as shown in equation Eq.S1 (Table 1-Note S1). Furthermore, the variance component of clones of the analysis without family effect ($\sigma_{c'}^2$) is estimated in a confounded way with the variance component of the family (Eq. S2).

$$\mathrm{MS}_{c'} = \frac{(s-1)\mathrm{MS}_s + s(c-1)\mathrm{MS}_c}{(s-1) + s(c-1)} \qquad \text{(Eq. S1)}$$

$$\sigma_{c'}^2 = \sigma_c^2 + \frac{c(s-1)\sigma_s^2}{sc-1} \qquad \text{(Eq. S2)}$$

In addition to the variance component of the clone, the empirical best linear unbiased prediction (BLUP) of clone effect ($BLUP_{c'}$) also is confounding with family effect. On the other hand, the BLUP of clone within-family effect ($BLUP_c$) is adjusted for family structure, removing confounding.

$$\mathrm{BLUP}_{c'} = \mathrm{h}_{c'}^2(\bar{\mathrm{y}}_{i\cdot} - \bar{\mathrm{y}}_{\cdot\cdot}) \qquad \text{(Eq. S3)}$$

$$\mathrm{BLUP}_c = \mathrm{h}_c^2\left[\bar{\mathrm{y}}_{i\cdot} - \bar{\mathrm{y}}_{\cdot\cdot} - \mathrm{h}_s^2\left(\bar{\mathrm{y}}_{\cdot j} - \bar{\mathrm{y}}_{\cdot\cdot}\right)\right] \qquad \text{(Eq. S4)}$$

$$\mathrm{BLUP}_s = \mathrm{h}_s^2\left(\bar{\mathrm{y}}_{\cdot j} - \bar{\mathrm{y}}_{\cdot\cdot}\right) \qquad \text{(Eq. S5)}$$

The ANOVA table and BLUP expressions previously scribed are suitable for an experiment established in a balanced complete block design. In other designs, such as incomplete block designs, the variance parameters, and BLUP are easily accounted for by using the residual maximum likelihood (REML) method (Patterson and Thompson, 1971) and Henderson's mixed model equations (Henderson et al., 1959).

Note S2. Confidence intervals for parameters of model METMpF

The 95% Chi-Squared confidence intervals were used for variance parameters of the unstructured matrices (**G**$_{tc'}$, **G**$_{ts}$, and **G**$_{tc}$) as shown in equations $\mathrm{Eq.S6}$, $\mathrm{Eq.S8}$, and $\mathrm{Eq.S10}$, respectively (SAS Institute Inc, 2016).

$$\frac{\nu_{c'}\hat{\sigma}^2_{c'_j}}{\chi_{\nu_{c'},1-\alpha/2}} \leq \sigma^2_{c'_j} \leq \frac{\nu_{c'}\hat{\sigma}^2_{c'_j}}{\chi_{\nu_{c'},\alpha/2}} \qquad (\mathrm{Eq.S6})$$

$$\nu_{c'} = 2\left(\frac{\hat{\sigma}^2_{c'_j}}{\sqrt{\nabla^{-1}_{AIc'j}}}\right)^2 \qquad (\mathrm{Eq.S7})$$

$$\frac{\nu_{s}\hat{\sigma}^2_{s_j}}{\chi_{\nu_{s},1-\alpha/2}} \leq \sigma^2_{s_j} \leq \frac{\nu_{s}\hat{\sigma}^2_{s_j}}{\chi_{\nu_{s},\alpha/2}} \qquad (\mathrm{Eq.S8})$$

$$\nu_{s} = 2\left(\frac{\hat{\sigma}^2_{s_j}}{\sqrt{\nabla^{-1}_{AIsj}}}\right)^2 \qquad (\mathrm{Eq.S9})$$

$$\frac{\nu_{c}\hat{\sigma}^2_{c_j}}{\chi_{\nu_{c},1-\alpha/2}} \leq \sigma^2_{c_j} \leq \frac{\nu_{c}\hat{\sigma}^2_{c_j}}{\chi_{\nu_{c},\alpha/2}} \qquad (\mathrm{Eq.S10})$$

$$\nu_{c} = 2\left(\frac{\hat{\sigma}^2_{c_j}}{\sqrt{\nabla^{-1}_{AIcj}}}\right)^2 \qquad (\mathrm{Eq.S11})$$

where: $\nu_{c'}$, ν_s, and ν_c are the Satterthwaite's degrees of freedom for the variance estimates of clone, family, and clone within-family effects; $\hat{\sigma}^2_{c'_j}$, $\hat{\sigma}^2_{s_j}$, and $\hat{\sigma}^2_{c_j}$ are the variance estimates of clone, family, and clone within-family effects; $\chi^2_{\nu,1-\alpha/2}$ and $\chi^2_{\nu,\alpha/2}$ are the upper and lower percentiles which enclose (1-α) of a χ^2 distribution with ν degrees of freedom; $\nabla^{-1}_{AIc'j}$, ∇^{-1}_{AIsj}, and ∇^{-1}_{AIcj} are the asymptotic variances of variance estimates of clone, family, and clone within-family effects, extracted from diagonal of inverse of average information matrix in the last iteration.

For genotypic correlations between pairs of environments for the clone, family, and clone within-family effects, the 95% confidence intervals were based on Standard Normal distribution (Meyer, 2008).

$$\hat{\rho}_{G_{c'ij}} \pm z_{\alpha/2} SE_{c'ij} \qquad \text{(Eq. S12)}$$

$$\hat{\rho}_{G_{sij}} \pm z_{\alpha/2} SE_{sij} \qquad \text{(Eq. S13)}$$

$$\hat{\rho}_{G_{cij}} \pm z_{\alpha/2} SE_{cij} \qquad \text{(Eq. S14)}$$

where: $\hat{\rho}_{G_{c'ij}}$, $\hat{\rho}_{G_{sij}}$ and $\hat{\rho}_{G_{cij}}$ are estimates of genotypic correlations between pairs of environments for clone, family, and clone within-family effects, obtained via estimators (10), (11) and (12) described in section 2.2.2. of the Material and Methods, respectively; $z_{\alpha/2}$ is the $100(1\text{-}\alpha/2)^{th}$ percentile of a standard normal distribution; $SE_{c'ij}$, SE_{sij}, and SE_{cij} are standard errors for clone, family, and clone within-family effects.

The standard errors of genotypic correlations required in equations Eq. S12, Eq. S13, and Eq. S14 were obtained by the delta method (Masuda, 2019; Gold et al., 2020), as shown in equations Eq. S15, Eq. S17, and Eq. S19.

$$SE_{c'ij} = \sqrt{\theta_{c'}^{T} \nabla_{AIc'}^{-1} \theta_{c'}} \qquad \text{(Eq. S15)}$$

$$\theta_{c'}^{T} = \begin{bmatrix} \frac{\partial \rho_{G_{c'ij}}}{\partial \sigma_{c'_{ij}}} & \frac{\partial \rho_{G_{c'ij}}}{\partial \sigma_{c'_{i}}^{2}} & \frac{\partial \rho_{G_{c'ij}}}{\partial \sigma_{c'_{j}}^{2}} \end{bmatrix} \qquad \text{(Eq. S16)}$$

$$SE_{sij} = \sqrt{\theta_{s}^{T} \nabla_{AIs}^{-1} \theta_{s}} \qquad \text{(Eq. S17)}$$

$$\theta_{s}^{T} = \begin{bmatrix} \frac{\partial \rho_{G_{sij}}}{\partial \sigma_{s_{ij}}} & \frac{\partial \rho_{G_{sij}}}{\partial \sigma_{s_{i}}^{2}} & \frac{\partial \rho_{G_{sij}}}{\partial \sigma_{s_{j}}^{2}} \end{bmatrix} \qquad \text{(Eq. S18)}$$

$$SE_{cij} = \sqrt{\theta_{c}^{T} \nabla_{AIc}^{-1} \theta_{c}} \qquad \text{(Eq. S19)}$$

$$\theta_{c}^{T} = \begin{bmatrix} \frac{\partial \rho_{G_{cij}}}{\partial \sigma_{c_{ij}}} & \frac{\partial \rho_{G_{cij}}}{\partial \sigma_{c_{i}}^{2}} & \frac{\partial \rho_{G_{cij}}}{\partial \sigma_{c_{j}}^{2}} \end{bmatrix} \qquad \text{(Eq. S20)}$$

where: $\theta_{c'}$, θ_{s} and θ_{c} are vectors of derivatives of genotypic correlations between pairs of environments for clone, family and clone within-family effects; $\nabla_{AIc'}^{-1}$,∇_{AIs}^{-1}, and ∇_{AIc}^{-1} are inverse of submatrices (associated with the parameters of genotypic correlations) of average information matrix.

2. **Supplementary tables**

Table S1. Summary of the variance components for the single trial model without family effect (STMwF), and single trial model plus family effect (STMpF) for traits total tuber yield (TTY, Mg ha^{-1}), and specific gravity [SG ($\times 10^{-6}$)] in different seasons.

Trait	Trial†	STMwF‡			STMpF‡			
		σ_b^2	$\sigma_{c'}^2$	σ^2	σ_b^2	σ_s^2	σ_c^2	σ^2
TTY	POP1(WHS)	5.14	78.84	108.44	3.96	13.86	66.48	108.84
	POP2(WHS)	7.80	47.31	43.90	5.52	11.60	35.69	47.56
	POP2(MHS)	5.91	57.55	89.01	5.75	6.75	49.72	90.20
	POP2(HHS)	0.18	57.73	25.09	0.16	3.63	54.18	25.06
	POP3(WHS)	11.81	128.05	25.87	12.08	3.85	124.57	25.80
	POP3(HHS)	2.09	131.72	58.65	3.24	16.79	115.57	58.96
SG	POP1(WHS)	7.30	52.20	15.20	5.80	14.10	40.30	15.63
	POP2(WHS)	12.30	33.80	50.50	4.90	14.70	27.70	50.00
	POP2(MHS)	1.60	33.10	38.90	1.50	9.90	21.70	39.50
	POP2(HHS)	3.80	54.90	38.00	3.50	12.60	42.30	38.40
	POP3(WHS)	7.60	134.00	90.70	8.00	15.80	117.00	93.40
	POP3(HHS)	7.20	52.40	29.90	6.80	4.20	47.50	30.70

†The trial identification: POP1(WHS), POP2(WHS), POP2(MHS), POP2(HHS), POP3(WHS), and POP3(HHS), where the codes POP1, POP2, and POP3 identify the different clonal population and codes WHS, MHS, and HHS identify three different seasons, varying in the function of stress level: without heat stress (WHS), moderate heat stress (MHS), and high heat stress (HHS).
$\sigma_b^2, \sigma_s^2, \sigma_{c'}^2, \sigma_c^2$,and σ^2: variance components associated with block, family, clone, clone within-family, and error effects.

Table S2. The overall mean of clonal populations for the single trial model without family effect (STMwF) and single trial model plus family effect (STMpF) for trait total tuber yield (TTY, Mg ha^{-1}) and specific gravity (SG) in different seasons.

Trait	Trials†	STMwF	STMpF
TTY	POP1(WHS)	39.18	39.29
	POP2(WHS)	25.77	25.61
	POP2(MHS)	30.11	29.98
	POP2(HHS)	14.94	14.96
	POP3(WHS)	24.92	24.79
	POP3(HHS)	24.23	24.50
SG	POP1(WHS)	1.080	1.081
	POP2(WHS)	1.088	1.088
	POP2(MHS)	1.067	1.068
	POP2(HHS)	1.057	1.057
	POP3(WHS)	1.069	1.069
	POP3(HHS)	1.060	1.060

†The trial identification: POP1(WHS), POP2(WHS), POP2(MHS), POP2(HHS), POP3(WHS), and POP3(HHS), where the codes POP1, POP2, and POP3 identify the different clonal population and codes WHS, MHS, and HHS identify three different seasons, varying in the function of stress level: without heat stress (WHS), moderate heat stress (MHS), and high heat stress (HHS).

Table S3. Czekanowski coefficient (CC) and Spearman correlation coefficient (r_S) between the vector of clone effects ($\mathbf{u}_{c'}$) from the single trial model without family effect (STMwF) and the vector of total genotypic effects of clones ($\mathbf{u}_{gST}$) from the single trial model plus family effect (STMpF) for traits total tuber yield (TTY, Mg ha^{-1}), and specific gravity (SG) in different seasons.

Trial†	TTY		SG	
	CC	r_S	CC	r_S
POP1(WHS)	0.88	0.95^{**}	0.87	0.98^{**}
POP2(WHS)	0.80	0.92^{**}	0.71	0.83^{**}
POP2(MHS)	0.89	0.97^{**}	0.74	0.91^{**}
POP2(HHS)	0.97	0.99^{**}	0.81	0.93^{**}
POP3(WHS)	1.00	1.00^{**}	0.91	0.98^{**}
POP3(HHS)	0.95	0.99^{**}	0.92	0.99^{**}

†The trial identification: POP1(WHS), POP2(WHS), POP2(MHS), POP2(HHS), POP3(WHS), and POP3(HHS), where the codes POP1, POP2, and POP3 identify the different clonal population and codes WHS, MHS, and HHS identify three different seasons, varying in the function of stress level: without heat stress (WHS), moderate heat stress (MHS), and high heat stress (HHS).
Significance of Spearman correlation coefficient, p-value < 0.01 '**'.

Table S4. Czekanowski coefficient (CC) and Spearman correlation coefficient (r_S) between the vector of clone effects ($\mathbf{u}_{c}$) from single trial model plus family effect (STMpF) and the vector of total genotypic effects of clones ($\mathbf{u}_{gST}$) from single trial model plus family effect (STMpF) for traits total tuber yield (TTY, Mg ha^{-1}), and specific gravity (SG) in different seasons.

Trial†	TTY		SG	
	CC	r_S	CC	r_S
POP1(WHS)	0.78	0.87^{**}	0.73	0.85^{**}
POP2(WHS)	0.69	0.82^{**}	0.61	0.72^{**}
POP2(MHS)	0.82	0.92^{**}	0.56	0.75^{**}
POP2(HHS)	0.90	0.96^{**}	0.70	0.83^{**}
POP3(WHS)	0.91	0.99^{**}	0.76	0.89^{**}
POP3(HHS)	0.89	0.93^{**}	0.82	0.96^{**}

†The trial identification: POP1(WHS), POP2(WHS), POP2(MHS), POP2(HHS), POP3(WHS), and POP3(HHS), where the codes POP1, POP2, and POP3 identify the different clonal population and codes WHS, MHS, and HHS identify three different seasons, varying in the function of stress level: without heat stress (WHS), moderate heat stress (MHS), and high heat stress (HHS).
Significance of Spearman correlation coefficient, p-value < 0.01 '**'.

Table S5. Summary of the multi-environment trial model without family effect (METMwF) and multi-environment trial model plus family effect (METMpF): Maximum point of the residual log-likelihood (ℓ) and Akaike information criterion (AIC).

Trait‡	Population†	Models	ℓ	AIC
TTY	POP2	METMwF	-4218.37	8460.74
		METMpF	-4203.85	8443.69
SG	POP2	METMwF	6336.45	-12648.90
		METMpF	6363.00	-12690.00
	POP3	METMwF	-1116.97	2247.95
		METMpF	-1116.69	2253.37

‡Total tuber yield (TTY, Mg ha^{-1}) and specific gravity (SG).
†Clonal populations POP2 and POP3.

Table S6. Summary of genetic and non-genetic variance parameters estimates for the multi-environment trial model without family effect (METMwF) from clonal populations POP2. Lower confidence limit (LCL) and upper confidence limit (UCL) of the 95% confidence interval for variance parameters associated with the traits total tuber yield (TTY, Mg ha^{-1}) and specific gravity (SG).

Parameters†	TTY			SG ($\times 10^6$)		
	LCL	Estimates	UCL	LCL	Estimates	UCL
$\sigma^2_{b_{\mathrm{WHS}}}$	4.31	8.73	26.21	6.37	12.20	32.20
$\sigma^2_{b_{\mathrm{MHS}}}$	1.86	4.90	32.53	0.30	1.09	37.40
$\sigma^2_{b_{\mathrm{HHS}}}$				1.46	3.69	20.80
$\sigma^2_{c'_{\mathrm{WHS}}}$	31.20	48.08	83.64	16.10	32.20	93.60
$\sigma^2_{c'_{\mathrm{MHS}}}$	30.50	58.31	152.95	17.50	30.70	67.50
$\sigma^2_{c'_{\mathrm{HHS}}}$	44.87	58.18	78.48	37.60	54.50	86.10
$\sigma^2_{e_{\mathrm{WHS}}}$	28.25	43.01	73.35	34.20	52.20	89.40
$\sigma^2_{e_{\mathrm{MHS}}}$	58.69	88.88	150.32	26.20	39.50	66.40
$\sigma^2_{e_{\mathrm{HHS}}}$	16.63	24.91	41.39	25.40	38.60	65.60
$\rho_{G_{c'_{\mathrm{WHS,MHS}}}}$	0.19	0.45	0.71	0.35	0.77	1.20
$\rho_{G_{c'_{\mathrm{WHS,HHS}}}}$	-0.05	0.11	0.27	0.23	0.52	0.82
$\rho_{G_{c'_{\mathrm{MHS,HHS}}}}$	0.14	0.36	0.58	0.37	0.65	0.92

†$\sigma^2_{b_{\mathrm{WHS}}}$, $\sigma^2_{b_{\mathrm{MHS}}}$, $\sigma^2_{b_{\mathrm{HHS}}}$, $\sigma^2_{c'_{\mathrm{WHS}}}$, $\sigma^2_{c'_{\mathrm{MHS}}}$, $\sigma^2_{c'_{\mathrm{HHS}}}$, $\sigma^2_{e_{\mathrm{WHS}}}$, $\sigma^2_{e_{\mathrm{MHS}}}$ and $\sigma^2_{e_{\mathrm{HHS}}}$- Variance components associated with effects of block, clone within-family, and error for the trials WHS, MHS, and HHS, respectively; $\rho_{G_{c'_{\mathrm{WHS,MHS}}}}$, $\rho_{G_{c'_{\mathrm{WHS,HHS}}}}$, and $\rho_{G_{c'_{\mathrm{MHS,HHS}}}}$- The genotypic correlations between pairs of environments for clone within-family ($\rho_{G_{c'}}$) effects for the trials WHS, MHS, and HHS, respectively.

Table S7. Summary of genetic and non-genetic variance parameters estimates for multi-environment trial model plus family effect (METMpF) from clonal populations POP2. Lower confidence limit (LCL) and upper confidence limit (UCL) of the 95% confidence interval for variance parameters associated with the traits total tuber yield (TTY, Mg ha^{-1}) and specific gravity (SG).

Parameters†	TTY			SG ($\times 10^6$)		
	LCL	Estimates	UCL	LCL	Estimates	UCL
$\sigma^2_{b_{\mathrm{WHS}}}$	2.33	5.83	32.30	2.40	6.17	37.30
$\sigma^2_{b_{\mathrm{MHS}}}$	1.68	4.57	34.33	0.31	1.10	36.90
$\sigma^2_{b_{\mathrm{HHS}}}$				1.32	3.38	20.40
$\sigma^2_{s_{\mathrm{WHS}}}$	5.61	11.32	33.65	6.57	13.10	37.70
$\sigma^2_{s_{\mathrm{MHS}}}$	3.01	7.32	36.66	5.40	9.99	24.40
$\sigma^2_{s_{\mathrm{HHS}}}$	1.35	3.64	26.53	6.43	12.40	33.10
$\sigma^2_{c_{\mathrm{WHS}}}$	20.55	36.63	82.97	12.50	27.20	98.00
$\sigma^2_{c_{\mathrm{MHS}}}$	24.12	50.34	162.44	10.10	21.50	72.30
$\sigma^2_{c_{\mathrm{HHS}}}$	41.66	54.62	74.79	27.50	43.00	76.70
$\sigma^2_{e_{\mathrm{WHS}}}$	30.55	46.71	80.25	26.40	39.80	66.90
$\sigma^2_{e_{\mathrm{MHS}}}$	59.11	89.94	152.03	32.90	50.50	87.30
$\sigma^2_{e_{\mathrm{HHS}}}$	16.62	24.88	41.32	25.50	38.70	65.70
$\rho_{G_{s_{\mathrm{WHS,MHS}}}}$	-0.74	-0.03	0.68	0.15	0.56	0.98
$\rho_{G_{s_{\mathrm{WHS,HHS}}}}$	-0.69	0.07	0.83	0.03	0.50	0.98
$\rho_{G_{s_{\mathrm{MHS,HHS}}}}$	0.02	0.69	1.36	0.54	0.81	1.09
$\rho_{G_{c_{\mathrm{WHS,MHS}}}}$	0.19	0.54	0.89	0.24	0.79	1.34
$\rho_{G_{c_{\mathrm{WHS,HHS}}}}$	-0.05	0.14	0.33	0.13	0.44	0.76
$\rho_{G_{c_{\mathrm{MHS,HHS}}}}$	0.09	0.33	0.57	0.23	0.59	0.95

† $\sigma^2_{b_{\mathrm{WHS}}}$, $\sigma^2_{b_{\mathrm{MHS}}}$, $\sigma^2_{b_{\mathrm{HHS}}}$, $\sigma^2_{s_{\mathrm{WHS}}}$, $\sigma^2_{s_{\mathrm{MHS}}}$, $\sigma^2_{s_{\mathrm{HHS}}}$, $\sigma^2_{c_{\mathrm{WHS}}}$, $\sigma^2_{c_{\mathrm{MHS}}}$, $\sigma^2_{c_{\mathrm{HHS}}}$, $\sigma^2_{e_{\mathrm{WHS}}}$, $\sigma^2_{e_{\mathrm{MHS}}}$ and $\sigma^2_{e_{\mathrm{HHS}}}$ - Variance components associated with effects of block, family, clone within-family, and error for the trials WHS, MHS, and HHS, respectively; $\rho_{G_{s_{\mathrm{WHS,MHS}}}}$, $\rho_{G_{s_{\mathrm{WHS,HHS}}}}$, $\rho_{G_{s_{\mathrm{MHS,HHS}}}}$, $\rho_{G_{c_{\mathrm{WHS,MHS}}}}$, $\rho_{G_{c_{\mathrm{WHS,HHS}}}}$, and $\rho_{G_{c_{\mathrm{MHS,HHS}}}}$ - The genotypic correlations between pairs of environments for family (ρ_{G_s}), and clone within-family (ρ_{G_c}) effects for the trials WHS, MHS, and HHS, respectively.

Table S8. Summary of factor analysis for traits total tuber yield (TTY, Mg ha^{-1}) and specific gravity (SG), estimated from the multi-environment trial model without family effect (METMwF) and multi-environment trial model plus family effect (METMpF), in different seasons.

Strategies	Trait	Trial†	Factor 1	Factor 2	Factor 3	Communities
$\mathbf{u}_c$	TTY	POP2(WHS)	0.03	-0.96	-0.01	0.92
		POP2(MHS)	-0.06	-0.88	-0.33	0.89
		POP2(HHS)	-0.08	-0.20	-0.96	0.98
	SG	POP2(WHS)	-0.95	0.02	0.05	0.91
		POP2(MHS)	-0.98	-0.02	0.00	0.97
		POP2(HHS)	-0.86	-0.03	-0.22	0.79
		Average				0.91
$\mathbf{u}_{c'}$	TTY	POP2(WHS)	0.05	-0.96	0.02	0.92
		POP2(MHS)	-0.04	-0.79	-0.47	0.85
		POP2(HHS)	-0.08	-0.15	-0.97	0.96
	SG	POP2(WHS)	-0.95	0.05	0.02	0.91
		POP2(MHS)	-0.98	0.00	-0.01	0.96
		POP2(HHS)	-0.90	-0.01	-0.17	0.84
		Average				0.91
$\mathbf{u}_{gMET}$	TTY	POP2(WHS)	-0.06	-0.95	-0.02	0.91
		POP2(MHS)	0.00	-0.70	-0.55	0.80
		POP2(HHS)	0.07	-0.11	-0.96	0.94
	SG	POP2(WHS)	0.90	0.13	0.07	0.83
		POP2(MHS)	0.97	-0.03	-0.02	0.94
		POP2(HHS)	0.88	-0.01	-0.19	0.82
		Average				0.87

†The trial identification: POP2(WHS), POP2(MHS), and POP2(HHS), where the code POP2 identifies the clonal population and codes WHS, MHS, and HHS identify three different seasons, varying in the function of stress level: without heat stress (WHS), moderate heat stress (MHS), and high heat stress (HHS).

3. Supplementary references

Gold, A., Olin, N., and Wang, A. (2020). What is the Delta Method? Available online at: www.cran.r-project.org/web/packages/modmarg/vignettes/delta-method.html (accessed August 10, 2022).

Henderson, C. R., Kempthorne, O., Searle, S. R., and von Krosigk, C. M. (1959). The Estimation of Environmental and Genetic Trends from Records Subject to Culling. *Biometrics* 15, 192. doi: 10.2307/2527669.

Masuda, Y. (2019). Variance component estimation. Available online at: www.masuday.github.io/blupf90_tutorial/vc_aireml.html (accessed August 12, 2022).

Meyer, K. (2008). Likelihood calculations to evaluate experimental designs to estimate genetic variances. *Heredity (Edinb)* 101, 212–221. doi: 10.1038/hdy.2008.46.

Patterson, H. D., and Thompson, R. (1971). Recovery of inter-block information when block sizes are unequal. *Biometrika* 58, 545–554. doi: 10.1093/biomet/58.3.545.

Piepho, H. P., Möhring, J., Melchinger, A. E., and Büchse, A. (2008). BLUP for phenotypic selection in plant breeding and variety testing. *Euphytica* 161, 209–228. doi: 10.1007/s10681-007-9449-8.

Resende, M. D. V., Ramalho, M. A. P., Carneiro, P. C. S., Carneiro, J. E. S., Batista, L. G., and Gois, I. B. (2016). Selection Index with Parents, Populations, Progenies, and Generations Effects in Autogamous Plant Breeding. *Crop Sci* 56, 530–546. doi: 10.2135/cropsci2015.05.0303.

SAS Institute Inc (2016). SAS/STAT® 14.2 User's Guide. *Software* (Cary, NC: SAS Institute Inc.).
